# Hypocapnia in acute carbon monoxide poisoning: A complex interaction beyond metabolic compensation

**DOI:** 10.1371/journal.pone.0356796

**Published:** 2026-09-08

**Authors:** Yongai Ling, Xianwei Xiong, Changsheng Ye, Weiguang Wang

**Affiliations:** 1 Health Promotion Center, People’s Hospital of Anji, Huzhou, Zhejiang, China; 2 Department of Emergency Medicine, People’s Hospital of Anji, Huzhou, Zhejiang, China; 3 General Practice Department, People’s Hospital of Anji, Huzhou, Zhejiang, China; University of Alcala Faculty of Medicine and Health Sciences: Universidad de Alcala Facultad de Medicina y Ciencias de la Salud, SPAIN

## Abstract

**Introduction:**

Acute carbon monoxide poisoning frequently induces complex acid-base disturbances; however, whether hypocapnia serves solely as a compensatory response to metabolic acidosis or involves additional factors remains insufficiently characterized.

**Methods:**

We retrospectively analyzed 940 emergency department patients with acute carbon monoxide poisoning (January 2019–February 2025), stratified by base excess into an acidosis group (<−3 mmol/L) and a non-acidosis group (≥ −3 mmol/L). Group comparisons used Mann-Whitney U tests, Spearman’s correlations assessed bivariate relationships, and hierarchical regression modeled PaCO_2_ determinants.

**Results:**

The acidosis group (*n* = 169) showed higher carboxyhemoglobin (26.5% vs. 21.6%) and lactate (4.4 vs. 1.7 mmol/L), and lower pH (7.37 vs. 7.41) and PaCO_2_ (33 vs. 39 mmHg) (all *P* < 0.001). Base excess correlated positively with PaCO_2_ (*ρ* = 0.489) and pH (*ρ* = 0.498), and negatively with lactate (*ρ* = −0.414) (all *P* < 0.001). Base excess was the strongest independent predictor of PaCO_2_ (*β* = 0.866), with each 1 mmol/L decrease associated with a 1.57 mmHg reduction (95% CI: 1.47–1.68). These associations were more pronounced in acidotic patients (*β* = 1.289 vs. 0.453) and males (*β* = 1.106 vs. 0.750). Curve-fitting showed only a modest linear relationship (*R*^2^ = 0.211).

**Conclusion:**

In acute carbon monoxide poisoning, metabolic acidosis is associated with hypocapnia, but the relationship is modest and context-dependent. The limited linear association between base excess and PaCO_2_, together with effect modification by acidosis status and sex, indicates hypocapnia involves factors beyond simple metabolic compensation.

## Introduction

Acute carbon monoxide poisoning, resulting from excessive inhalation of carbon monoxide, is a significant global cause of occupational and environmental poisoning-related morbidity and mortality. Elevated risks persist even years after exposure [1–3]. This condition is characterized by complex acid-base disturbances, most commonly presenting as concurrent metabolic acidosis and primary respiratory alkalosis. Importantly, the severity of respiratory alkalosis has been shown to have a dose-dependent association with adverse cardiovascular outcomes [4,5]. Substantial experimental and clinical evidence demonstrates that the associated hypocapnia contributes to multiorgan dysfunction, particularly involving the central nervous, cardiovascular, and pulmonary systems, through multiple synergistic effects [6–9].

A longitudinal study demonstrated a high prevalence of hypocapnia upon emergency department presentation (131/194, 67.5%; median PaCO_2_: 32.9 [29.5–36.4] mmHg), with persistent hypocapnia observed in a substantial proportion of patients (117/194, 60.3%; median PaCO_2_: 33.0 [31.0–36.7] mmHg) even six hours after admission, by which time carboxyhemoglobin levels had normalized. This sustained reduction in PaCO_2_, occurring after the presumed resolution of the primary hypoxic insult, challenges the hypothesis that hypocapnia is solely a compensatory response to concurrent metabolic acidosis [4].

The observed temporal dissociation between carboxyhemoglobin clearance and persistent hypocapnia underscores a gap in understanding the primary drivers of hypocapnia in acute carbon monoxide poisoning. Moreover, the full spectrum of acid-base disturbances, particularly the clinically significant occurrences of inappropriately normal PaCO_2_ or overt hypercapnia in the setting of severe metabolic acidosis, remains inadequately characterized. To address these limitations, this study aimed to systematically examine the relationship between PaCO_2_ and markers of metabolic acidosis across the entire clinical spectrum of patients with acute carbon monoxide poisoning. We specifically sought to determine whether hypocapnia functions predominantly as a compensatory response, or whether its presence and magnitude are modulated by additional, independent pathophysiological factors, including those that may attenuate the expected ventilatory compensation.

## Methods

### Patient identification and enrollment

We retrospectively identified consecutive patients presenting to emergency departments with suspected acute carbon monoxide poisoning between January 2019 and February 2025 by querying the information management systems of regional general hospitals, using “carbon monoxide poisoning” as the primary diagnostic search term. The data were accessed for research purposes between March 1, 2025, and April 10, 2025.

A standardized data collection protocol was employed to systematically capture the following variables for each eligible case: demographic characteristics (age, sex), unique identifiers (outpatient and national ID numbers), and comprehensive clinical parameters obtained from both initial emergency department evaluations and 30-day follow-up records.

The data collection process included detailed documentation of exposure history, clinical presentation, comorbidities, working diagnosis, clinical measurements (including arterial blood gases, troponin I levels, and hepatic and renal function tests), neuroimaging findings (head CT scans), and emergency interventions. Diagnostic confirmation was performed through a structured, four-phase protocol: (1) electronic screening based on predefined inclusion criteria to identify potential cases; (2) blinded review of medical charts by two independent physicians to confirm preliminary diagnoses; (3) multimodal diagnostic validation incorporating biochemical, radiologic, and clinical response assessments; and (4) institutional data reconciliation to eliminate duplicate records from intra- and inter-hospital transfers, thereby ensuring the uniqueness of each case in the final study cohort.

### Inclusion and exclusion criteria

This study investigates non-occupational, unintentional carbon monoxide poisoning originating from domestic sources, patients were considered eligible if they fulfilled all three diagnostic criteria for acute carbon monoxide poisoning: (1) documented exposure to carbon monoxide (e.g., from charcoal or coal heating systems, gas water heaters, or other combustion sources); (2) presence of characteristic neurological symptoms (including dizziness, headache, confusion, syncope, lethargy, delirium, or coma); and (3) laboratory confirmation by arterial blood gas analysis demonstrating a carboxyhemoglobin concentration ≥ 10%, consistent with the Chinese diagnostic criteria for mild intoxication (GBZ23–2024).

The exclusion criteria were defined as follows: (1) age under 14 years, due to legal restrictions on pediatric research; (2) cases involving inhalation of fire-related fumes or vehicle exhaust, intentional self-harm, or exposure to other complex toxicants such as cyanide, all of which are associated with distinct high-concentration exposure patterns; (3) concurrent diagnosis of acute cerebrovascular events, to minimize potential neurological confounding, or pre-existing severe psychiatric disorders, due to the possibility of comorbid interactions; and (4) absence of arterial blood gas analysis.

### Data collection and clinical variable definitions

Demographic information (age, sex) and clinical parameters were systematically collected, including arterial blood gas measurements (pH, PaCO_2_, lactate, standard base excess, carboxyhemoglobin) and serum biomarkers (hemoglobin, cardiac troponin I, creatinine, alanine aminotransferase, aspartate aminotransferase). Arterial blood gas parameters, including carboxyhemoglobin concentration, were measured using the ABL90 FLEX blood gas analyzer (Radiometer Medical ApS, Brønshøj, Denmark), which employs co-oximetry for direct spectrophotometric measurement of carboxyhemoglobin. Standard base excess was automatically calculated by the blood gas analyzer using the Siggaard-Andersen equation, which incorporates hemoglobin concentration for correction. Owing to the limitations inherent in the retrospective study design, complete data on electrolytes, albumin, and phosphate were unavailable for all patients and thus were not included in the formal analysis. During and after data collection, the authors did not have access to information that could identify individual participants, as all patient identifiers (including outpatient and national ID numbers) were anonymized prior to analysis.

Metabolic acidosis, the primary outcome of interest, was operationally defined as a standard base excess < −3 mmol/L, in accordance with the established clinical threshold. The normal reference range for standard base excess was specified as −3 to +3 mmol/L. Based on this criterion, patients were categorized into either the metabolic acidosis group (standard base excess < −3 mmol/L) or the non-acidosis group (≥ −3 mmol/L). The reference intervals applied in this study were as follows: arterial pH 7.35–7.45, PaCO_2_ 35–45 mmHg, lactate < 1.6 mmol/L, and carboxyhemoglobin < 2% for non-smokers. In line with these reference intervals, hypocapnia, normocapnia, and hypercapnia were defined as PaCO_2_ < 35 mmHg, 35–45 mmHg, and > 45 mmHg, respectively.

### Ethics approval and consent to participate

This retrospective study using anonymized clinical data was approved by the Ethics Committee of People’s Hospital of Anji (Approval No. P20230902-1/20230902-1-2) in compliance with the Declaration of Helsinki. Due to the retrospective nature of the study and the use of anonymized data, the requirement for informed consent was waived by the ethics committee.

### Methodological approaches for bias control and data quality assurance

This study employed three strategies to minimize potential biases: (1) using a carboxyhemoglobin concentration ≥ 10% as an inclusion criterion to minimize confounding by smoking, as smoking-related carboxyhemoglobin levels generally remain below 10%; (2) including patients with hepatorenal insufficiency to maintain cohort representativeness despite small sample size; and (3) excluding early deaths and minimally symptomatic cases, as they were unlikely to provide relevant data on metabolic acidosis. Although exposure duration was unknown, admission carboxyhemoglobin concentrations confirmed acute exposure and indicated poisoning severity. All primary blood gas data were available. The methodology follows established standards in observational toxicology and supports the validity of the main outcome.

### Statistical analysis

Statistical analyses were conducted using SPSS 26.0 (IBM Corp.). Non-normally distributed continuous variables were presented as median and interquartile range (IQR) and were compared between groups using the Mann-Whitney U test. Spearman’s rank-order correlation was employed to assess the associations between PaCO_2_ and metabolic parameters, including base excess, lactate, and pH. Multivariable linear regression models were constructed in a stepwise manner with PaCO_2_ as the dependent variable. Model 1 assessed base excess and lactate without adjustment; Model 2 adjusted for pH; and Model 3 added an interaction term between base excess and lactate. In the regression results, zero-order correlations denote simple bivariate associations; partial correlations reflect the association between each predictor and PaCO_2_ after adjusting for all other predictors; and part (semi-partial) correlations represent the unique contribution of each predictor beyond the others in the model. Model fit was evaluated using adjusted *R²* and F-tests. Subgroup analyses were performed according to the presence of metabolic acidosis, control status, and sex. The base excess–PaCO_2_ relationship was visualized using scatterplots, LOESS curves (bandwidth = 0.8), and second- and third-order polynomial fits. All analyses used complete-case data, two-tailed tests (*α* = 0.05), and results are reported with 95% confidence intervals for β-coefficients.

## Results

### Study population, group characteristics, and intergroup comparisons

**Fig 1:**
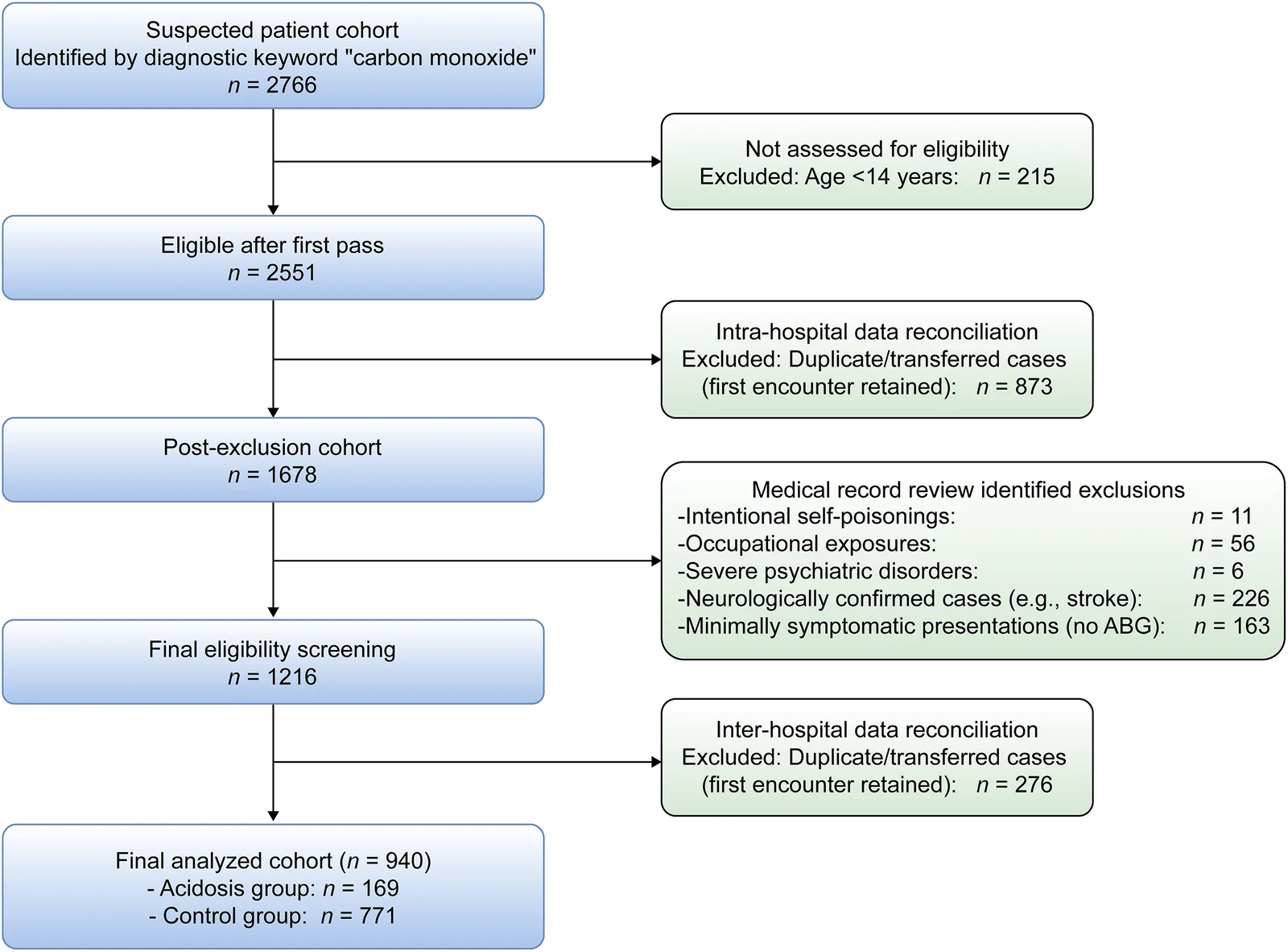
Flowchart of patient enrollment and exclusion. A total of 2,766 potential cases were screened from the electronic health record system. After excluding 215 patients under 14 years of age, 462 ineligible cases, and 1,149 duplicate or transfer records, 940 patients were included in the final analysis. Patients were categorized into metabolic acidosis group (base excess < –3 mmol/L, *n* = 169) and non-acidosis group (base excess ≥ –3 mmol/L, *n* = 771).

From 2,766 potential cases identified through electronic health record screening, we excluded 215 patients under 14 years of age, 462 ineligible cases (including self-poisoning, occupational exposure, psychiatric disorders, acute cerebrovascular events, and minimally symptomatic cases without arterial blood gas confirmation), and 1,149 duplicate or transfer cases, resulting in a final cohort of 940 patients (mean age 54.7 ± 19.0 years; 350 males [37.2%]). Participants were categorized according to arterial blood gas parameters into a metabolic acidosis group (standard base excess < −3 mmol/L, *n* = 169) and a non-acidosis group (*n* = 771), as shown in Fig 1.

Patients with acidosis were significantly older and had markedly higher carboxyhemoglobin concentrations (26.5% vs. 21.6%) and lactate levels (4.4 vs. 1.7 mmol/L), but lower arterial pH (7.37 vs. 7.41) and PaCO_2_ (33 vs. 39 mmHg) compared to the non-acidosis group (all *P* < 0.001; Table 1). PaCO_2_ distribution differed across base excess groups (S1 Table). Compared to the normal group (72.7% normocapnia, 21.7% hypocapnia, 5.6% hypercapnia), the low group was characterized by predominant hypocapnia (67.6%) and a lower rate of normocapnia (29.5%), although five patients (2.89%) with metabolic acidosis and severe consciousness impairment presented with respiratory acidosis and elevated PaCO_2_. The high group presented a unique pattern, with normocapnia remaining most frequent (61.2%) but with equal and notable shares of hypocapnia and hypercapnia (19.4% each).

**Table 1:** Comparison of clinical parameters between acidosis and non-acidosis groups.

Variable	Acidosis group (*n* = 169)	Non-acidosis group (*n* = 771)	Test statistic (*U*)	*P*-value
Age, years	60 (43–75)	55 (38–69)	74299	0.004
Arterial pH	7.37 (7.33–7.40)	7.41 (7.39–7.44)	26532	<0.001
PaCO_2_, mmHg	33 (28.8–35.2)	39 (35.6–41.5)	25957.5	<0.001
Lactate, mmol/L	4.4 (2.5–7.4)	1.7 (1.2–2.3)	107643.5	<0.001
Carboxyhemoglobin concentration, %	26.5 (18.8–35.5)	21.6 (15.3–28.1)	84205	<0.001

Note. Data are presented as median (IQR). Group comparisons were performed using the Mann‑Whitney U test.

### Associations between metabolic parameters and PaCO_2_: Correlation and multivariate regression analyses

Spearman correlation analysis confirmed significant bivariate associations among key metabolic parameters in the acute carbon monoxide poisoning cohort (all *P* < 0.001). Base excess showed moderate positive correlations with PaCO_2_ (*r* = 0.489) and pH (*r* = 0.498), and a moderate negative correlation with lactate (*r* = −0.414). PaCO_2_ was negatively correlated with lactate (*r* = −0.394) and pH (*r* = −0.287), with the latter being weaker. The weakest correlation was observed between lactate and pH (*r* = −0.132) (Table 2).

**Table 2:** Spearman correlation coefficient matrix for blood gas parameters.

Variable	PaCO_2_	Base excess	Lactate	Arterial pH
PaCO_2_	1.000	0.489**	−0.394**	−0.287**
Base excess	0.489**	1.000	−0.414**	0.498**
Lactate	−0.394**	−0.414**	1.000	−0.132**
Arterial pH	−0.287**	0.498**	−0.132**	1.000

Note. Spearman’s rank correlation coefficients (*ρ*) are shown. **P < 0.01 (two-tailed). The diagonal represents self-correlation (1.000).

To evaluate the independent contributions of these variables to PaCO_2_ variability, hierarchical regression analyses were performed. In Model 1, which included base excess and lactate without adjustment, both variables were significant predictors. In Model 2, with the addition of pH, the model fit was substantially improved (*F* = 567.316, *P* < 0.001; _Δ_*R*^2^ = 0.426). Model 3, which added an interaction term between base excess and lactate, yielded only negligible improvement (_Δ_*R*^2^ = 0.001, *P* = 0.097), supporting Model 2 as the most parsimonious and optimal specification. The following results are therefore derived from Model 2.

**Fig 2:**
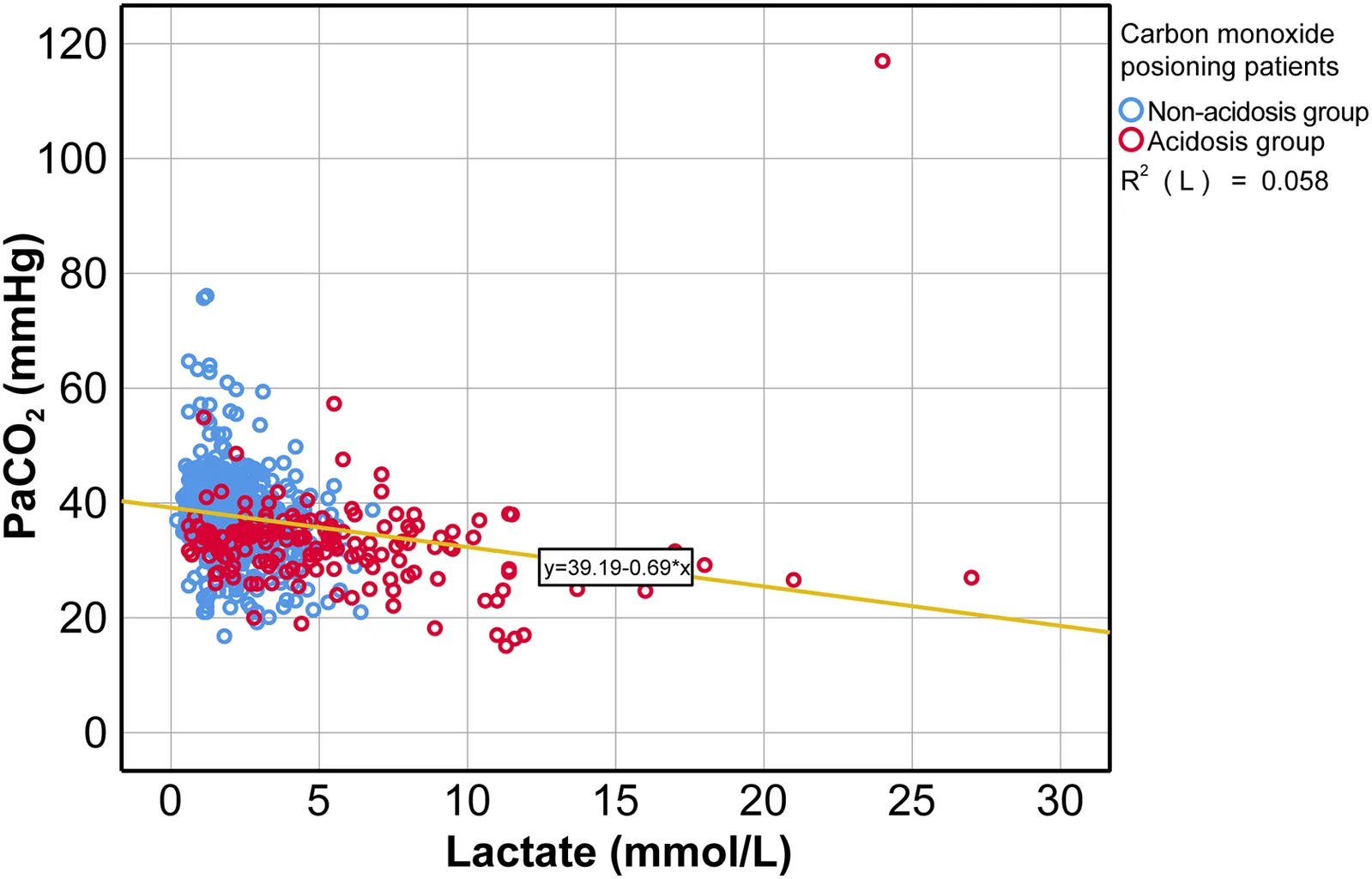
Scatter plot showing the relationship between lactate and PaCO_2_. A weak bivariate association was observed (*R*^2^ = 0.058), indicating that lactate alone explains only a limited proportion of the variance in PaCO_2_.

In this model, base excess emerged as the strongest independent predictor of PaCO_2_ (standardized *β* = 0.866), with its effect strengthened after adjustment (zero-order *r* = 0.459; part correlation = 0.556; partial correlation = 0.682). Despite a weak zero-order correlation (*r* = −0.257), pH demonstrated a robust independent effect (standardized *β* = −0.809; part correlation = −0.652; partial correlation = −0.738). Lactate showed a minimal independent association with PaCO_2_ (standardized *β* = −0.106; partial *r* = −0.130), a finding further supported by its weak bivariate relationship (Fig 2; *R*^2^ = 0.058).

The interaction term in Model 3 approached but did not reach statistical significance for model improvement (*P* = 0.097), indicating no substantial synergistic effect between lactate and base excess. Complete regression results are presented in Tables 3 and S2 and S3 Tables.

**Table 3:** Multiple linear regression analysis predicting PaCO_2_ (Model 2).

Predictor	Unstandardized Coefficients		Standardized Coefficients	*t*	*P*-value	95% CI for *B*		Correlations			Model Summary	
	*B*	Std. Error	*β*			Lower Bound	Upper Bound	Zero- order	Partial	Part	Adj.*R*^*2*^	*F*
Constant	704.1	19.859		35.455	<0.001	665.126	743.073				0.644	567.316
Base excess	1.573	0.055	0.866	28.550	<0.001	1.465	1.681	0.459	0.682	0.556		
Lactate	−0.293	0.073	−0.106	−3.998	<0.001	−0.437	−0.149	−0.381	−0.130	−0.078		
Arterial pH	−89.709	2.678	−0.809	−33.504	<0.001	−94.963	−84.454	−0.257	−0.738	−0.652		

Note. Dependent variable: PaCO_2_ (mm Hg). The unstandardized coefficient (B) for base excess can be interpreted as the change in PaCO_2_ (mmHg) per 1 mmol/L change in base excess. The standardized coefficient (β) indicates the relative importance of each predictor within the model.

### Differential respiratory compensation patterns in acidosis vs non-acidosis groups

A stratified analysis was conducted to assess whether the association between base excess and PaCO_2_ differed by metabolic acidosis status, specifically through comparison of regression coefficients (*β*) between groups. The slope was markedly steeper in the acidosis group (standardized *β* = 1.289) than in the non-acidosis group (standardized *β* = 0.453). The effect of pH also differed by group, showing a 54% stronger inverse association in the acidosis group (standardized *β* = −1.097) compared to the non-acidosis group (standardized *β* = −0.713). Lactate was not significantly associated with PaCO_2_ in the acidosis group (*P* = 0.453), likely due to uniformly elevated levels, whereas a weak negative association was observed in the non-acidosis group (standardized *β* = −0.095) (Table 4).

**Table 4:** Multiple linear regression analysis predicting PaCO_2_ stratified by acidosis status.

Group	Predictor	Unstandardized Coefficients		Standardized Coefficients	*t*	*P*-value	95% CI for *B*		Correlations			Model Summary	
		*B*	Std. Error	*β*			Lower Bound	Upper Bound	Zero- order	Partial	Part	Adj.*R*^*2*^	*F*
Acidosis (*n* = 169)	Constant	715.57	38.507		18.583	<0.001	639.553	791.586				0.723	150.87
	Base excess	1.952	0.109	1.289	17.861	<0.001	1.736	2.168	0.476	0.809	0.716		
	Lactate	−0.059	0.079	−0.040	−0.753	0.453	−0.214	0.096	−0.350	−0.058	−0.030		
	Arterial pH	−91.061	5.170	−1.097	−17.615	<0.001	−101.266	−80.856	−0.091	−0.805	−0.707		
Non-acidosis (*n* = 771)	Constant	710.07	23.221		30.578	<0.001	664.483	755.655				0.582	353.923
	Base excess	1.463	0.081	0.453	18.069	<0.001	1.304	1.622	0.281	0.549	0.424		
	Lactate	−0.576	0.147	−0.095	−3.910	<0.001	−0.865	−0.287	−0.270	−0.141	−0.092		
	Arterial pH	−90.439	3.137	−0.713	−28.832	<0.001	−96.596	−84.281	−0.603	−0.723	−0.676		

Note. Abbreviations as in Table 3.

### Sex modulates acid-base compensation patterns in carbon monoxide poisoning

Sex-stratified analyses confirmed statistically significant associations between base excess and PaCO_2_ in both male and female cohorts (all *P* < 0.001), with males exhibiting a greater effect size for base excess (standardized *β* = 1.106, 95% CI: 1.611–1.917 vs. standardized *β* = 0.750, 95% CI: 1.344–1.635) and a larger impact of pH (standardized *β* = −0.920, 95% CI: −111.43 to −95.237 vs. standardized *β* = −0.747, 95% CI: −90.302 to −76.683). Lactate demonstrated sex-specific associations, revealing no significant relationship in males (*P* = 0.116), while showing a weak negative association in females (standardized *β* = −0.129, 95% CI: −0.595 to −0.195). These findings support the robustness of the primary analysis and highlight clinically relevant sex differences in acid-base regulatory relationships (Table 5).

**Table 5:** Multiple linear regression analysis predicting PaCO_2_ stratified by sex.

Group	Predictor	Unstandardized Coefficients		Standardized Coefficients	*t*	*P*-value	95% CI for *B*		Correlations			Model Summary	
*B*	Std. Error	*β*	Lower Bound	Upper Bound	Zero- order	Partial	Part	Adj.*R*^*2*^	*F*
Female (*n* = 690)	Constant	658.01	25.731		25.573	<0.001	607.469	708.540				0.595	289.602
	Base excess	1.490	0.074	0.750	20.083	<0.001	1.344	1.635	0.429	0.639	0.527		
	Lactate	−0.395	0.102	−0.129	−3.883	<0.001	−.595	−0.195	−0.358	−0.158	−0.102		
	Arterial pH	−83.493	3.467	−0.747	−24.081	<0.001	−90.302	−76.683	−0.307	−0.705	−0.631		
Male (*n* = 350)	Constant	805.02	30.489		26.403	<0.001	705.049	864.984				0.747	343.826
	Base excess	1.764	0.078	1.106	22.623	<0.001	1.611	1.917	0.532	0.772	0.610		
	Lactate	−0.156	0.099	−0.065	−1.577	0.116	−.352	0.039	−0.459	−0.084	−0.043		
	Arterial pH	−103.334	4.117	−0.920	−25.102	<0.001	−111.43	−95.237	−0.142	−0.803	−0.676		

Note. Abbreviations as in Table 3.

### Nonlinear analysis reveals limited linear association between base excess and PaCO_2_

**Fig 3:**
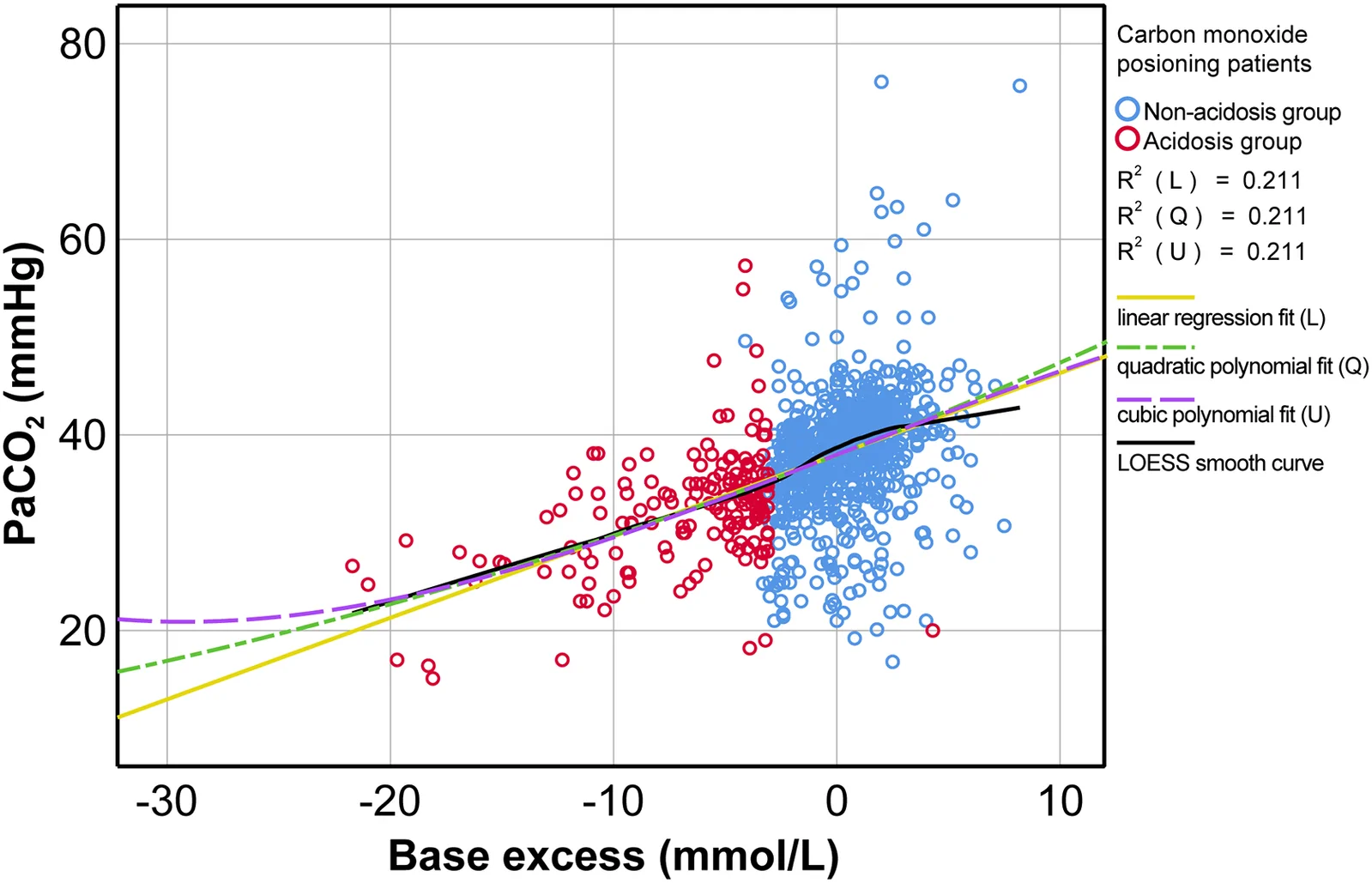
Relationship between base excess and PaCO_2_. Blue dots represent patients without metabolic acidosis (base excess ≥ –3 mmol/L), and red dots represent patients with metabolic acidosis (base excess < –3 mmol/L). Four fitted curves are shown: yellow-brown solid line, linear regression fit; green dashed line, quadratic polynomial fit; purple dotted line, cubic polynomial fit; and black solid line, LOESS smooth curve. The comparable R^2^ values across polynomial models and the close alignment of the LOESS curve with the linear fit indicate that the association between base excess and PaCO_2_ is predominantly linear, with negligible nonlinear components.

Scatter plots and model-fitting techniques were used to evaluate the linearity of the primary association. A moderate linear relationship was observed (*R*^2^ = 0.211). The LOESS smooth curve closely followed the linear regression line across most of the data range, and adding quadratic and cubic polynomial terms did not meaningfully improve model fit (_Δ_*R*^2^ < 0.01 for both). These findings indicate a statistically significant but limited linear trend (*P* < 0.001 for the linear model), implying that unmeasured physiological or clinical factors substantially influence PaCO_2_ regulation (Fig 3).

## Discussion

Carbon monoxide exhibits dose-dependent duality, acting as a cytoprotective signaling molecule at low concentrations but causing multiorgan damage via hypoxia, oxidative stress, and immune-inflammatory activation upon acute high-dose exposure [10–12]. Its core toxicity stems from a 200- to 400-fold greater affinity for hemoglobin than oxygen [10,13,14], which impairs oxygen delivery and initiates a cascade of cellular injury. This includes sustained mitochondrial dysfunction and neuroinflammatory pathways involving nitric oxide and reactive oxygen species [13,15–17].

This pathophysiological cascade underlies the characteristic acid-base disturbances in acute carbon monoxide poisoning: tissue hypoxia and mitochondrial dysfunction synergistically drive acidosis, which in turn triggers hyperventilation through the activation of central or peripheral chemoreceptors, or as a compensatory response to the metabolic acidosis itself, ultimately resulting in respiratory alkalosis. Consequently, acid-base disturbances constitute a defining feature, primarily manifesting as a combination of metabolic acidosis and respiratory alkalosis. A similar pattern of metabolic acidosis with compensatory hypocapnia has also been observed in other toxicological contexts, such as acetone poisoning [18]. Clinical investigations have established this dual acid-base imbalance as an independent predictor of in-hospital mortality [19]. This pathophysiological profile has been consistently documented across swine, canine, and rodent models of carbon monoxide exposure [20–23]. Population-based cohort studies further reveal that 62.2% of patients develop concurrent small airway obstruction and mixed ventilatory dysfunction [24].

The classical acid-base compensation paradigm proposes that metabolic acidosis initiates an immediate respiratory compensatory mechanism through hyperventilation-mediated CO_2_ elimination, resulting in a rapid decrease in PaCO_2_ to maintain the HCO_3_^-^/PaCO_2_ ratio. In contrast, renal compensation generally requires several hours to days to reach full physiological effect [25–27]. In the context of carbon monoxide poisoning, conventional pathophysiology attributes the acid-base disturbance to lactate accumulation caused by tissue hypoxia, leading to metabolic acidosis [21,28,29]. Contrastingly, recent clinical evidence demonstrates that primary respiratory alkalosis is the most common initial acid-base abnormality, often emerging prior to the development of measurable metabolic acidosis [4,22].

Although respiratory alkalosis represents the most prevalent acid-base disturbance in critically ill patients, with its severity showing a direct, pH-independent association with adverse clinical outcomes independent of concurrent metabolic acidosis, the underlying pathophysiological features exhibit distinct characteristics in the context of carbon monoxide poisoning. In this condition, hypocapnia originates from hyperventilation induced by heightened respiratory drive, which is significantly associated with both serum lactate concentrations and poisoning severity [30–33]. This ventilatory response is predominantly mediated by central chemoreceptor activation in response to hydrogen ion (H⁺) accumulation during acidotic states [13,30,34–36]. The resultant hypocapnia contributes to complex cerebral injury via three principal pathways: (1) vasoconstriction primarily involving the cerebellar and putamen regions [32,37–39]; (2) metabolic dysregulation characterized by increased glucose consumption and impaired excitatory-inhibitory synaptic balance [6,7], and (3) heightened susceptibility to periventricular leukomalacia and intraventricular hemorrhage [40]. In our study, the bivariate analysis demonstrated a moderate negative correlation between lactate and PaCO_2_ (*ρ* = –0.394); however, this association was substantially attenuated in the multivariate regression model after adjustment for base excess and pH. This discrepancy suggests that lactate is primarily a downstream byproduct of metabolic acidosis rather than a direct respiratory stimulant. The minimal independent effect of lactate on PaCO_2_ in our model suggests that the bivariate correlation was largely explained for by acid-base status rather than a direct effect of lactate on respiratory drive.

This study demonstrated that concomitant respiratory distress at admission, along with key arterial blood gas parameters such as carboxyhemoglobin concentration, lactate concentration, and base excess, were independently associated with adverse clinical outcomes [41]. To investigate the underlying basis of these clinical observations, we examined the relationships among key blood gas parameters. Our analysis revealed that patients with metabolic acidosis were older and had higher levels of carboxyhemoglobin and lactate, but lower pH and PaCO_2_, compared to those without. Spearman correlations confirmed that decreasing base excess correlated with lower pH and PaCO_2_ and higher lactate. This finding is consistent with previous studies that have also reported a positive association between lactate and carboxyhemoglobin [42,43]. The inverse relationships between PaCO_2_ and both pH and lactate suggest active respiratory compensation. Multivariate regression showed the lactate–PaCO_2_ association was largely mediated by base excess, indicating lactate is a secondary metabolic byproduct, not a primary respiratory stimulus. Although lactate may stimulate respiration during cerebral hypoxia, its overall effect in lactic acidosis appears depressant [35].

Our preliminary multivariate analysis quantified the stoichiometry of respiratory compensation for metabolic acid-base disturbances. Both lower base excess and lower pH were independently associated with lower PaCO_2_, with standardized coefficients of *β* = 0.866 and *β* = –0.809, respectively. The association with base excess strengthened after adjusting for covariates, highlighting its distinct and robust contribution. Specifically, the model estimates a 1.573 mmHg decrease in PaCO_2_ per 1 mmol/L reduction in base excess and a 8.97 mmHg decrease per 0.1-unit drop in pH, consistent with established compensatory physiology. To assess whether this relationship remains consistent across clinical contexts, we conducted stratified analyses. These revealed a state-dependent gain: among patients with metabolic acidosis (base excess < –3 mmol/L), the standardized slope linking base excess to PaCO_2_ (*β* = 1.289) was 2.8-fold steeper than in those without acidosis (*β* = 0.453). This indicates markedly enhanced ventilatory sensitivity to metabolic acid load under acidic conditions.

However, this compensatory mechanism is influential but not deterministic. In the final multivariable linear regression model (Model 2), base excess explained only 21.1% of PaCO_2_ variance (*R*^2^ = 0.211), with negligible improvement from nonlinear modeling (_Δ_*R*^2^ < 0.01). The combination of a context-modulated response (state-dependent gain) and inherently limited explanatory power provides strong quantitative support for the “complex interaction” central to our thesis. It demonstrates that the final PaCO_2_ level reflects an integration of compensatory drives and other independent ventilatory inputs, rather than being dictated by metabolic status alone.

Additional clinical subgroups support these findings. Five patients with metabolic acidosis and severe consciousness impairment had respiratory acidosis with elevated PaCO_2_, deviating from typical compensatory hypocapnia. This suggests that profound central nervous system depression can impair ventilatory drive, leading to compensation failure. Gender analysis showed a 48% stronger association in males than females (*β* = 1.106 vs. 0.750), indicating sex-specific regulation of respiratory control, possibly due to differences in gas exchange or carbon monoxide clearance in men [44]. Collectively, these results show a continuum from effective compensation to decompensation and demonstrate that the base excess–PaCO_2_ relationship is dynamically influenced by neurologic status, sex, and other factors. However, lack of electrolyte and albumin data limited use of anion gap or Stewart methods, potentially missing key acid-base determinants.

The pathophysiological mechanisms underlying hypocapnia have been explored in prior animal studies and mathematical modeling, which suggest that hyperventilation following carbon monoxide exposure may result from activation of central chemoreceptors secondary to cerebral hypoxia and cerebrospinal fluid acidosis [35,36]. A recent study demonstrated that carbon monoxide bound to neuroglobin serves as a functional reservoir, potentially prolonging neuronal injury following exposure [10]. This finding points to an ongoing source of central nervous system stimulation beyond the initial hypoxic insult. In light of this finding, we propose that the hypocapnia observed in our cohort, particularly when present in the absence of significant metabolic acidosis, is likely driven in part by direct central nervous system stimulation by carbon monoxide or by cerebral hypoxia, rather than representing a passive compensatory response to systemic acidemia alone. This hypothesis is supported by our quantitative and subgroup findings, and aligns with clinical observations of persistent hypocapnia after carboxyhemoglobin levels have normalized [4], indicating that respiratory drive can be sustained independently of metabolic acid-base status.

These insights underscore the necessity for a nuanced clinical approach to hypocapnia. Its interpretation must be context-dependent: hypocapnia reflects appropriate respiratory compensation in the context of metabolic acidosis, may signify non-metabolic stimuli such as pain, anxiety, or central nervous system activation when acidosis is absent, and should prompt concern for potential respiratory depression when missing despite established acidosis. Moreover, significant hypocapnia itself poses clinical risks, including cerebral vasoconstriction and cardiac impairment. Therefore, management should prioritize correction of the underlying hypoxia and acid–base disturbances. When enhanced ventilation is indicated, maintaining normocapnia, particularly through monitored isocapnic hyperventilation, offers a strategic advantage by accelerating carbon monoxide elimination while minimizing the adverse physiological consequences of low PaCO_2_ [45,46].

It is important to note that this was a retrospective observational study, which can establish associations but not causal relationships. Further prospective studies incorporating direct measurements of VCO_2_, cardiac output, and minute ventilation are necessary to elucidate the underlying physiological mechanisms.

### Limitations

This study has several limitations. First, excluding minimally symptomatic cases without blood gas analysis and occupational exposures may limit generalizability. Second, missing data on exposure duration, prehospital oxygen use, and unmeasured confounders could introduce potential bias. Third, although a ≥ 10% carboxyhemoglobin threshold was used to reduce smoking-related bias, variability in sampling timing may have affected severity classification. While regression models explained much of the variance, residual confounding remains possible. Subgroup analyses (e.g., by sex or acidosis status) should be interpreted cautiously due to low statistical power. The cross-sectional design prevents causal inference. Prospective studies with serial monitoring are needed to clarify the temporal dynamics of hypocapnia during intoxication and recovery.

## Conclusions

This retrospective study characterized the metabolic and respiratory disturbances associated with acute carbon monoxide poisoning. Patients presenting with metabolic acidosis exhibited significantly elevated carboxyhemoglobin concentrations and lactate concentrations, along with decreased pH and PaCO_2_. Although base excess strongly predicted PaCO_2_, curve-fitting showed only a modest linear association, unchanged by higher-order polynomial models, indicating that hypocapnia involves more than simple metabolic compensation. These results highlight the complex interaction between acid-base balance and respiratory control, with notable sex-specific differences in compensatory responses.

## Supporting information

S1 Table Distribution of PaCO_2_ levels across base excess groups. (DOC)

S2 Table Multiple linear regression analysis predicting PaCO_2_ (Model 1). (DOC)

S3 Table Multiple linear regression analysis predicting PaCO_2_ (Model 3). (DOC)
