# Supplementary material for: Hypocapnia in acute carbon monoxide poisoning: A complex interaction beyond metabolic compensation
Source: PLoS One. 2026 Sep 8;21(9):e0356796. doi: 10.1371/journal.pone.0356796 (PMC13552748; doi:10.1371/journal.pone.0356796)
Supplement: Supplementary file 1 [file pone.0356796.s001.doc]

S1 Table. Distribution of PaCO₂ levels across base excess groups

| Base excess group | PaCO_2_ 35-45mmHg (*n*, %) | PaCO_2_ < 35 mmHg (*n*, %) | PaCO_2_ > 45mmHg (*n*, %) | Total |
| --- | --- | --- | --- | --- |
| Normal base excess  (-3 to 3mmol/L) | 509 (72.71%) | 152 (21.71%) | 39 (5.57%) | 700 |
| Low base excess (< -3mmol/L) | 51 (29.48%) | 117 (67.63%) | 5 (2.89%) | 173 |
| High base excess (> 3mmol/L) | 41 (61.19%) | 13 (19.40%) | 13 (19.40%) | 67 |
| Total | 601 | 282 | 57 | 940 |

Note. Data are presented as number of patients (percentage within each base excess group).
