# Supplementary material for: Hypocapnia in acute carbon monoxide poisoning: A complex interaction beyond metabolic compensation
Source: PLoS One. 2026 Sep 8;21(9):e0356796. doi: 10.1371/journal.pone.0356796 (PMC13552748; doi:10.1371/journal.pone.0356796)
Supplement: Supplementary file 2 [file pone.0356796.s002.doc]

S2 Table. Multiple linear regression analysis predicting PaCO₂ (Model 1)

| Predictor | Unstandardized Coefficients | | Standardized Coefficients | *t* | *P*-value | 95% CI for *B* | | Correlations | | | Model Summary | |
| --- | --- | --- | --- | --- | --- | --- | --- | --- | --- | --- | --- | --- |
|  | *B* | Std. Error | *β* |  |  | Lower Bound | Upper Bound | Zero-  order | Partial | Part | Adj.*R^2^* | *F* |
| Constant | 38.711 | 0.310 | — | 125.26 | <0.001 | 38.164 | 39.379 |  |  |  | 0.218 | 131.864 |
| Base excess | 0.675 | 0.071 | 0.371 | 9.461 | <0.001 | 0.535 | 0.815 | 0.459 | 0.295 | 0.273 |  |  |
| Lactate | -0.357 | 0.109 | -0.129 | -3.288 | 0.001 | -0.570 | -0.144 | -0.381 | -0.107 | -0.095 |  |  |

Note. Dependent variable: PaCO₂ (mm Hg). The unstandardized coefficient (B) for base excess can be interpreted as the change in PaCO₂ (mmHg) per 1 mmol/L change in base excess. The standardized coefficient (β) indicates the relative importance of each predictor within the model.
