# Supplementary material for: Hypocapnia in acute carbon monoxide poisoning: A complex interaction beyond metabolic compensation
Source: PLoS One. 2026 Sep 8;21(9):e0356796. doi: 10.1371/journal.pone.0356796 (PMC13552748; doi:10.1371/journal.pone.0356796)
Supplement: Supplementary file 3 [file pone.0356796.s003.doc]

S3 Table. Multiple linear regression analysis predicting PaCO₂ (Model 3)

| Predictor | Unstandardized Coefficients | | Standardized Coefficients | *t* | *P*-value | 95% CI for *B* | | | Correlations | | | | Model Summary | |
| --- | --- | --- | --- | --- | --- | --- | --- | --- | --- | --- | --- | --- | --- | --- |
|  | *B* | Std. Error | *β* |  |  | Lower Bound | | Upper Bound | Zero-  order | Partial | | Part | Adj.*R^2^* | *F* |
| Constant | 712.91 | 20.538 | — | 34.712 | <0.001 | 672.606 | | 753.217 |  |  | |  | 0.645 | 426.976 |
| Base excess | 1.531 | 0.061 | 0.842 | 25.283 | <0.001 | 1.412 | 1.650 | | 0.459 | | 0.637 | 0.492 |  |  |
| Lactate | -0.190 | 0.096 | -0.069 | -1.980 | 0.048 | -0.379 | -0.002 | | -0.381 | | -0.065 | -0.039 |  |  |
| Arterial pH | -90.923 | 2.773 | -0.820 | -32.787 | <0.001 | -96.365 | -85.481 | | -0.257 | | -0.731 | -0.638 |  |  |
| Base excess ×Lactate | 0.014 | 0.008 | 0.070 | 1.661 | 0.097 | -0.002 | 0.030 | | 0.330 | | 0.054 | 0.032 |  |  |

Note. Dependent variable: PaCO₂ (mm Hg). The unstandardized coefficient (B) for base excess can be interpreted as the change in PaCO₂ (mmHg) per 1 mmol/L change in base excess. The standardized coefficient (β) indicates the relative importance of each predictor within the model.
